# Effectiveness of Thermal Ablation for Renal Cell Carcinoma after Prior Partial Nephrectomy

**DOI:** 10.1016/j.euros.2023.08.005

**Published:** 2023-09-28

**Authors:** Mohamed E. Abdelsalam, Tessa N. Hudspeth, Laura Leonards, Samuel B. Kusin, Jennifer R. Buckley, Roland Bassett, Ahmed Awad, Jose A. Karam, Surena F. Matin, Thomas Lu, Kamran Ahrar

**Affiliations:** a Department of Interventional Radiology, The University of Texas MD Anderson Cancer Center, Houston, TX, USA; b Department of Radiology, Texas Radiology Associates, LLP, Plano, TX, USA; c Department of Radiology, North Oaks Medical Center, Hammond, LA, USA; d University of Texas Southwestern Medical Center, Dallas, TX, USA; e Department of Radiology, University of Missouri, Kansas City, MO, USA; f Department of Biostatistics, The University of Texas MD Anderson Cancer Center, Houston, TX, USA; g Department of Urology, The University of Texas MD Anderson Cancer Center, Houston, TX, USA

**Keywords:** Ablation, Recurrence, Partial nephrectomy

## Abstract

**Background:**

Repeat partial nephrectomy (PN) for tumors recurring in the ipsilateral kidney is associated with surgical complexity and a higher rate of complications.

**Objective:**

To evaluate the local oncologic efficacy of thermal ablation (TA) for renal cell carcinoma (RCC) in the ipsilateral kidney following PN.

**Design, setting, participation:**

We included patients who underwent ablation for renal tumors in the ipsilateral kidney after PN between January 2005 and December 2019. Demographics, tumor size, procedural details, complications, pathology, local oncologic outcomes, and survival outcomes are described.

**Outcome measurements and statistical analysis:**

The procedural, pathologic, and oncologic outcomes are described. Survival rates were estimated using the Kaplan-Meier method.

**Results and limitations:**

A total of 66 patients (46 male and 20 female) with a median age of 62 yr (interquartile range [IQR] 52–69) met our inclusion criteria. In these patients, 74 TA procedures were performed for 86 lesions (median tumor size 1.9 cm, IQR 1.6–2.5). Radiofrequency ablation and cryoablation accounted for 60 (81%) and 14 (19%) procedures, respectively. Three patients (3.7%) had Clavien-Dindo grade III complications. Of 65 lesion biopsies, 62 (95.5%) were diagnostic. The most common subtype was clear cell RCC (*n* = 37). The median imaging follow-up duration was 60 mo (IQR 43–88). Recurrence in the ablation zone occurred for four lesions (4.6%) at a median of 6.9 mo (IQR 6.4–10.7). The rates of overall, recurrence-free, and disease-free survival were 93.1%, 94.4%, and 65.6% at 5 yr, and 71.6%, 94.4%, and 60.1% at 10 yr, respectively. Limitations include the retrospective design and the lack of a control group.

**Conclusions:**

TA is effective for the treatment of RCC in the ipsilateral kidney following PN.

**Patient summary:**

Heat treatment to remove tumor tissue is an effective option for small kidney masses recurring after partial kidney removal for cancer. Long-term follow-up data revealed that this treatment resulted in low recurrence and complication rates.

## Take Home Message

Repeat partial nephrectomy for renal tumors recurring in the ipsilateral kidney is associated with greater surgical complexity and a higher rate of perioperative complications. Percutaneous ablation is an effective treatment option for these patients. Our data reveal long-term oncologic control with low recurrence and complication rates.

## 1 Introduction

Partial nephrectomy (PN) is the gold standard for management of small renal masses [1], [2]. In addition to favorable local tumor control and oncologic outcomes, PN has gained popularity over time because of its significant advantage in preservation of renal function in comparison to radical nephrectomy [1], [2], [3], [4]. In its most recent guidelines, the American Urological Association (AUA) recommends prioritizing PN for the management of cT1a renal cell carcinoma (RCC) and limiting radical nephrectomy to specific situations [1].

The rate of local tumor recurrence following PN is low (1.5–4.2%) [5], [6], [7]. However, such recurrences are associated with poor prognosis [8], [9], [10], [11], [12], [13]. Although some have suggested different options for the treatment of local recurrence, surgical extirpation is still considered the optimal option for such cases [14], [15]. Repeat PN is associated with greater surgical complexity secondary to postoperative desmoplasia, resulting in higher rates of perioperative complications and making renal preservation challenging in some cases [8], [15]. Therefore, alternative treatment options are needed for this population.

Over the past two decades, thermal ablation has gained wide acceptance as a management option for small renal masses [1], especially for older patients and those with comorbidities that are contraindications for surgical resection. Given the increasing evidence of favorable oncologic outcomes for thermal ablation of renal masses [16], [17], [18], [19], [20], [21], [22], [23], coupled with much better periprocedural outcomes and lower complication rates in comparison to PN [24], the AUA now considers thermal ablation an alternative management option for renal masses smaller than 3 cm [1]. Thermal ablation may be an effective treatment for patients with local renal cancer relapse following prior PN [25].

To the best of our knowledge, data on thermal ablation for the management of renal tumors in the ipsilateral kidney after PN are lacking. Therefore, we performed a retrospective study to determine the feasibility, safety, and local oncologic effectiveness of image-guided thermal ablation as a treatment option for renal tumors recurring in the ipsilateral kidney after PN.

## 2 Patients and methods

We performed a retrospective review of the renal ablation registry at our institution. The study was approved by our institutional review board and a waiver of informed consent was granted. All patients who underwent ablation for de novo renal masses in the ipsilateral kidney following PN over a 15-yr period (January 2005–December 2019) were considered for inclusion in the study. These patients were referred for ablation after multidisciplinary counseling by a treating urologist. Patients with de novo solitary renal tumors, recurrent renal tumors previously treated with ablation, recurrent tumors in the surgical bed following PN, or recurrent tumors in the contralateral kidney were excluded from the study. Our ablation technique has been described in detail previously [26], [27], [28]. In summary, we performed ablation procedures under general anesthesia using computed tomography (CT) or magnetic resonance imaging (MRI) guidance (when the lesion was not well-delineated on noncontrast CT). Depending on the size and location of the renal tumor, the decision is made to use radiofrequency ablation (RFA; Covidian, Mansfield, MA, USA) or percutaneous cryoablation (PCA; Endocare, HealthTronics, Austin, TX, USA).

Both RFA and PCA are equally effective in properly selected patients [29], [30], [31]. We perform RFA for lesions of ≤3.5 cm in size [32] in peripheral locations. For larger or central lesion, PCA is preferentially used [33]. Adjunctive techniques can be used to ensure safe ablation. We perform hydrodissection (mixture of 5% dextrose and nonionic contrast at a ratio of 60:1) to displace the surrounding at-risk structures.

When the ureteropelvic junction or ureter is at risk of thermal injury, we perform retrograde pyeloperfusion. A 5–6-F ureteral catheter is placed in the renal pelvis in a retrograde fashion. During RFA, cooled 5% dextrose is infused into the renal pelvis and drained via a Foley catheter in the bladder. During PCA, we use warm 5% dextrose or saline for infusion.

Multiphase contrast-enhanced cross-sectional imaging was performed at the end of the procedure to evaluate the adequacy of the ablation and its safety margin, as well as any immediate complications.

### 2.1 Data collection

The electronic medical record for each patient was reviewed, and the following information was recorded: demographic data (age and sex), history of prior renal cancer, previous renal cancer treatment, treatment modality (radical nephrectomy, PN, or ablation), tumor characteristics (size and side of renal lesions, relationship of new lesions to surgical margins), ablation procedure details (ablation only or biopsy and ablation, thermal ablation technology, imaging guidance modality, adjunctive techniques, technical success), complications (graded using the Clavien-Dindo classification system), pathologic outcome (RCC histology subtype and grade), duration of follow-up, and radiologic outcomes identified on follow-up imaging.

Contrast-enhanced follow-up imaging was performed at regular intervals for 24 mo and every 12 mo thereafter. All follow-up cross-sectional images were reviewed by two independent interventional radiologists to evaluate local oncologic outcomes, including identification and assessment of any residual or recurrent lesions at the ablation sites. In cases with discrepancy for the findings, a third interventional radiologist reviewed the case and a final decision was made among the three interventional radiologists.

### 2.2 Definitions

A residual tumor was defined as evidence of enhancement within the ablation zone in an imaging study at 1 or 3 mo after ablation. Definitions for oncologic and survival outcomes were adopted from the AUA guidelines on management of small renal masses [34]. Local recurrence was defined as new contrast uptake within the ablation zone not previously identified in the 1- and 3-mo follow-up imaging studies, or viable tumor cells identified in tissue samples from the ablation zone.

### 2.3 Data and statistical analysis

All demographic and lesion characteristics, ablation procedures, complications, and pathological outcomes were reported using an observational descriptive method. The Kaplan-Meier method was used to estimate overall survival (OS), local recurrence-free survival (RFS), and disease-free survival (DFS).

OS duration was measured from the date of the ablation procedure to date of death. RFS duration was measured from the date of the procedure to the date of local recurrence at the ablation site, performed on a per-lesion basis. DFS duration was measured from the date of the procedure to the date of any form of tumor recurrence at the ablation zone or in the kidney away from the ablation zone, or the development of distant metastatic disease.

## 3 Results

A total of 66 patients (46 male and 20 female) with a median age of 62 yr (interquartile range [IQR] 52–69, range 37–81) met the inclusion criteria. Twenty-three patients had a solitary kidney. We performed 74 ablation procedures for 86 lesions. The median tumor size was 1.9 cm (IQR 1.6–2.5, range 0.8–4.2, mean 2.07). The patient demographic data and lesion characteristics are summarized in Table 1.

**Table 1:** Demographic and tumor characteristics of the study population (*n* = 66)

Parameter	Result
Age (yr)	
Mean (standard deviation)	55 (11.0)
Median (range)	62 (37–81)
Sex, *n* (%)	
Female	20 (30.4)
Male	46 (69.6)
Tumor size (cm)	
Mean (standard deviation)	2.07 (0.70)
Median (range)	1.9 (0.8–4.2)
Biopsy outcome (*n* = 62), *n* (%)	
Renal cell carcinoma	59 (95)
Clear cell	37 (59.6)
Papillary	18 (9)
Chromophobe	1 (1.6)
Oncocytic variant	2 (3.2)
Not otherwise specified	1 (1.6)
Benign	3 (5)
Fuhrman grade, *n* (%)	
Grade 1	5 (9)
Grade 2	32 (54)
Grade 3	9 (15)
Not reported	13 (22)

### 3.1 Ablation procedures

Of the 74 ablation procedures, 66 (89%) were performed under CT guidance and eight (11%) under MRI guidance. RFA accounted for 60 (81%) and PCA for 14 (19%) procedures. Thirty-six and 38 ablation sessions involved the right and left kidney, respectively. Ablation was technically successful in all the patients. Hydrodissection and pyeloperfusion were successfully performed in 28 (38%) and two (2.7%) patients, respectively. Hydrodissection was used to displace the planned ablation zone from the colon (*n* = 14), duodenum (*n* = 2), or the psoas muscle/posterior abdominal wall (*n* = 12). The ablation technical success rate was 100%. Details of the ablation procedures are presented in Table 2.

**Table 2:** Ablation procedures and outcomes

Parameter	Lesions, *n* (%)
Kidney laterality	
Right	36 (48.5)
Left	38 (51.5)
Guidance modality	
CT	66 (90)
MRI	8 (10)
Ablation modality	
Cryoablation	14 (11)
RFA	60 (89)
Adjunctive technique	
Hydrodissection	28 (38)
Pyeloperfusion	2 (2.7)
Complications	
No	67 (90.6)
Yes	7 (9.4)

CT = computed tomography; MRI = magnetic resonance imaging; RFA = radiofrequency ablation.

### 3.2 Complications

Of the 74 ablations, seven (9.5%) had associated complications. According to the Clavien-Dindo classification, two patients had grade I, two had grade II, and three had grade III complications. Complications and their management are summarized in Table 3.

**Table 3:** Clavien-Dindo grade and management for ablation complications

Modality	SK	Complications	Management	Grade
PCA	Yes	Anuria, obstructing clot	Ureteral stent	III
PCA	No	Hematuria, obstructing clot	Percutaneous nephroureteral catheter	III
RFA	No	Urinary tract infection	Antibiotics	II
PCA	Yes	Ureteral injury, anuria	Ureteral stent and dialysis for hyperkalemia	III
RFA	No	Perirenal hematoma	Observation	I
RFA	No	Urinary tract infection	Antibiotic**s**	II
RFA	No	Urine leak	Observation	I

SK = solitary kidney; PCA = percutaneous cryoablation; RFA = radiofrequency ablation.

All grade III complications occurred after PCA. Two of the three patients with grade III complications had a solitary kidney. Both patients experienced ureteral obstruction that required temporary placement of a ureteral stent. The third patient experienced bleeding and obstruction of the ureter by a blood clot. The patient underwent percutaneous nephroureteral catheter placement. Ureteral obstruction was identified on the excretory phase of the immediate postablation multiphase contrast-enhanced CT as arrest of contrast flow at the level of the obstruction.

### 3.3 Pathologic outcomes

Renal biopsies were performed on 65 lesions and were diagnostic for 62 (59 RCCs and three benign lesions) and nondiagnostic for three lesions. Of the RCC cases, 37 (61%) were clear cell RCC, 18 (30.5%) were papillary RCC, one (1.7%) was chromophobe RCC, two (3.4%) were oncocytic-variant RCC, and two (3.4%) were RCC not otherwise specified. The Fuhrman grade was reported for 46 lesions, as summarized in Table 1.

### 3.4 Oncologic and survival outcomes

**Fig. 1:**
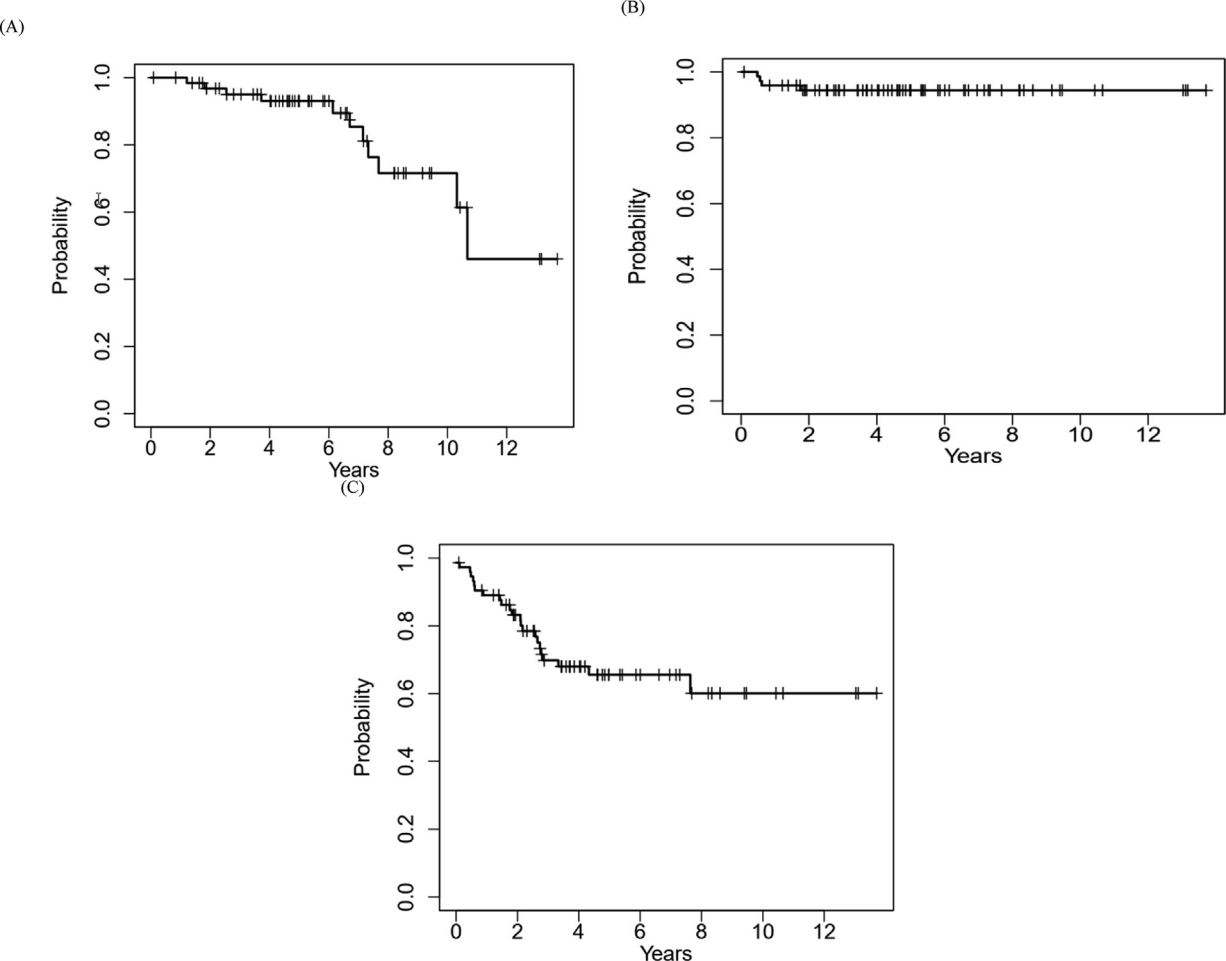
Kaplan-Meier curves for (A) overall survival, (B) recurrence-free survival, and (C) disease-free survival for the study population.

The median follow-up for this cohort was 60 mo (IQR 36.7–80.5, range 10–165, mean 66.8). None of the patients with ablated lesions had any residual disease immediately after ablation or within 3 mo of radiological follow-up. Local recurrence in the ablation zone occurred for four lesions (4.6%). Follow-up cross-sectional imaging identified all four recurrences at a median time to detection of 6.9 mo (IQR 6.4–10.7, range 5.7–21). All the recurrences followed RFA. Two of the recurrent lesions were central tumors, and two were mixed intraparenchymal/exophytic lesions. Biopsy confirmed three of the four recurrences before further treatment. The histological subtypes were clear cell RCC (*n* = 2) and papillary RCC (*n* = 1). For treatment of recurrence, two patients underwent RFA and two underwent surgical resection. Table 4 summarizes the tumor histology, time to detection, and management of local RCC recurrence. Thirteen patients had died by the time of data collection. Cause of death was documented in medical records for only three patients (heart attack, renal failure, and metastatic disease progression). The 5-yr survival rates were 93.1% (95% confidence interval [CI] 86.7–99.9%) for OS, 94.4% (95% CI 89.2–99.9%) for RFS, and 65.6% (95% CI 54.6–78.8%) for DFS, with corresponding 10-year rates of 71.6% (95% CI 71.6–91.4%), 94.4% (95% CI 89.2–99.9%), and 60.1% (95% CI 46.8–77.2%). Figure 1 shows Kaplan-Meier curves for OS, RFS, and DFS for the study cohort.

**Table 4:** Tumor histology, time to detection, and management of local recurrences following ablation

Case	SK	Tumor histology	Time to detection (mo)	Management
1	Yes	Clear cell renal cell carcinoma	5.7	Radiofrequency ablation
2	No	Clear cell renal cell carcinoma	6.6	Radical nephrectomy
3	Yes	Papillary renal cell carcinoma	7.3	Radical nephrectomy
4	No	Clear cell renal cell carcinoma	21.0	Radiofrequency ablation

SK = solitary kidney.

## 4 Discussion

This study demonstrated local oncologic efficacy of 94.7% and 5-yr RFS of 93.2% with percutaneous imaging-guided thermal ablation of renal tumors recurring after PN in the ipsilateral kidney. Recurrences in the ablation zone occurred in 5.3% of cases, with a median time to detection of 6.9 mo.

Morgan et al. [35] reviewed five patients who underwent cryoablation for locally recurrent renal tumors after ipsilateral PN. Two patients had Clavien-Dindo grade I complications. The median follow-up was 32 mo (range 20–39 mo). Local tumor recurrence occurred in one patient who underwent cryoablation. In a retrospective review by Hegg et al. [36] with a larger patient population, cryoablation was performed for 68 ipsilateral renal tumors after previous PN in 48 patients. Clavien-Dindo grade III or higher complications occurred in three patients. The median follow-up for patients with biopsy-proven or suspected RCCs (*n* = 54) was 19 mo (range 3–61 mo). Follow-up imaging revealed local tumor recurrence in five patients.

In the present study, we observed an ablation complication rate of 9.5%. This is in agreement with the rates of 8–20% reported in a systematic review of 82 studies involving patients with a solitary kidney who underwent renal ablation [37], [38] and lower than the rates following repeat PN in previous studies (range 19.6–52%) [11], [12], [13]. Our complication rate is comparable to the 5.7% reported by Hegg et al. [36] for renal ablation following ipsilateral PN in 48 patients. However, Morgan et al. [35] reported two grade I complications for six renal ablations (33%) following ipsilateral PN. This difference may be because of the large difference in the number of patients between these two studies (six vs 72).

Regarding the local oncologic effectiveness of imaging-guided thermal ablation for recurrent renal tumors in the ipsilateral kidney after PN, we found a recurrence rate of 5.3%. This is lower than recurrence rates reported following repeat PN in patients with almost the same follow-up duration [11], [12]. Specifically, over median follow-up of 56 mo, Johnson et al. [11] reported subsequent surgery for local recurrence or new lesions in 10/51 (20%) patients who underwent PN. Liu et al. [12] reported reoperation for either local recurrence or new lesions in eight of 22 patients (36%) at mean follow-up of 52 mo. Our local recurrence rate was not in concordance with the findings of Morgan et al. [35] or Hegg et al. [36], who reported local recurrence rates of 20% and 9.3%, respectively. The differences in local recurrence rates may be secondary to differences in the number of patients.

Limitations of our study include those inherent to its retrospective nature (eg, patient selection). Although the sample size is the largest to date on renal ablation in the ipsilateral kidney following PN, it remains relatively small. A larger series would help in better assessment of oncologic outcomes. In addition, only 73% of the lesions were biopsy-proven RCC before ablation. A study with 100% biopsy-proven RCC lesions would yield more conclusive results. Another limitation is that 13 patients had died by the time of data collection, but cause of death was documented in medical records for only three patients. Furthermore, this retrospective study had no control group to compare local ablation outcomes with those for surgery or active surveillance. However, our study yielded encouraging results regarding thermal ablation for recurrent renal lesions in the ipsilateral kidney after PN.

## 5 Conclusions

In conclusion, imaging-guided percutaneous thermal ablation is a safe and effective treatment option for renal tumors that recur in the ipsilateral kidney following PN. Long-term follow-up data revealed long-term oncologic control with low recurrence and complication rates. Although the ultimate decision regarding patient management should be individualized, given the present results, inclusion of imaging-guided percutaneous thermal ablation in the management algorithm for patients with renal tumors in the ipsilateral kidney after PN is reasonable.

  *Study concept and design*: Abdelsalam, Karam, Matin, Ahrar.

*Acquisition of data*: Abdelsalam, Hudspeth, Leonards, Kusin, Buckley, Awad, Lu.

*Analysis and interpretation of data*: Abdelsalam, Karam, Matin, Ahrar.

*Drafting of the manuscript*: Abdelsalam, Ahrar.

*Critical revision of the manuscript for important intellectual content*: Abdelsalam, Hudspeth, Leonards, Kusin, Buckley, Awad, Lu, Karam, Matin, Ahrar.

*Statistical analysis*: Bassett.

*Obtaining funding*: Abdelsalam.

*Administrative, technical, or material support*: None.

*Supervision*: Ahrar.

*Other*: None.

  ***Financial disclosures:*** Mohamed E. Abdelsalam certifies that all conflicts of interest, including specific financial interests and relationships and affiliations relevant to the subject matter or materials discussed in the manuscript (eg, employment/affiliation, grants or funding, consultancies, honoraria, stock ownership or options, expert testimony, royalties, or patents filed, received, or pending), are the following: None.

  ***Funding/Support and role of the sponsor:*** This work was supported in part by a National Institutes of Health/National Cancer Institute Cancer Center Support Grant (award number P30 CA016672) and the Biostatistics Resource Group. The sponsors played no direct role in the study.

  ***Data sharing statement:*** Data are available for bona fide researchers on request from the authors.
